# Alzheimer's disease: from molecular pathways to therapies

**DOI:** 10.1186/s43556-026-00443-2

**Published:** 2026-04-02

**Authors:** Jing-Qiu Feng, Ling-Ling Yang, Ya-Xi Luo, Xiu-Qing Yao

**Affiliations:** 1 Department of Rehabilitation, The Second Affiliated Hospital of Chongqing Medical University, Chongqing, China; 2 Chongqing Municipality Clinical Research Center for Geriatric Medicine, Chongqing, China; 3 Department of Rehabilitation Therapy, Chongqing Medical University, Chongqing, China; 4 College of Basic Medical Sciences, Naval Medical University, Shanghai, China

**Keywords:** Alzheimer disease, Therapy, Amyloid-β, Tau, Inflammation, Metabolic dysfunction

## Abstract

Alzheimer disease (AD) is the most common neurodegenerative disorder and a leading cause of dementia worldwide. With accelerating population aging, its incidence continues to rise, imposing a substantial burden on public health systems and society. Despite extensive advances in research, currently available therapies remain largely symptomatic and have limited capacity to halt or reverse disease progression. Recent progress in understanding the molecular and cellular mechanisms underlying AD has driven the development of targeted therapeutic strategies, particularly immunotherapies directed against amyloid-β (Aβ) and tau pathology. However, the pathogenesis of AD is highly complex and multifactorial, underscoring the need for a more integrated understanding of the interactions among diverse pathological processes and the identification of additional therapeutic targets. Here, we provide a systematic synthesis of the core pathological mechanisms of AD and their interconnected molecular pathways, together with a comprehensive overview of current targeted therapeutic strategies. We highlight recent advances in Aβ- and tau-directed immunotherapies and further examine emerging interventions targeting neuroinflammation, metabolic dysregulation, the gut microbiota, lifestyle-related factors, and neurogenesis, evaluating their potential based on evidence from both clinical and preclinical studies. By integrating mechanistic insights with therapeutic developments, this review outlines key opportunities and challenges in the evolving landscape of AD treatment. These perspectives may inform the development of next-generation disease-modifying therapies and contribute to a more comprehensive framework for understanding the pathogenesis and treatment of AD.

## Introduction

Alzheimer’s disease (AD) is an age-related, progressive neurodegenerative disorder and the most common cause of dementia, accounting for 60%–80% of all cases. With global population aging, the number of individuals affected by AD is projected to triple from approximately 50 million in 2019 to 2050, imposing substantial societal, economic, and caregiving burdens [1, 2]. Despite the growing prevalence of AD, no effective therapies are currently available. Medications used in clinical practice are primarily symptomatic. Agents that provide modest cognitive benefit include cholinesterase inhibitors (donepezil, galantamine, and rivastigmine) and the N-methyl-D-aspartate (NMDA) receptor antagonist memantine. Cholinesterase inhibitors increase acetylcholine (ACh) availability by preventing its breakdown in the synaptic cleft, thereby enhancing the efficiency of degenerating cholinergic neurons and providing modest improvement in symptoms, cognition, and daily function in patients with mild to moderate AD. Memantine, a low-affinity NMDA receptor antagonist, mitigates glutamate-mediated excitotoxicity while preserving normal physiological signaling and is used as an alternative symptomatic therapy for mild to severe AD. However, none of these drugs alter the course of the disease or modify its underlying pathophysiological mechanisms [3, 4].

Over the past decade, most investigational therapies for AD have failed in clinical trials, underscoring the scarcity of effective treatment options. Nevertheless, advances in understanding AD pathobiology have illuminated multiple molecular pathways, with antibody-based targeted therapies achieving notable progress in recent years [5, 6]. Given that extracellular amyloid-β (Aβ) plaque deposition and intracellular neurofibrillary tangles (NFTs) formation represent the defining pathological hallmarks of AD, current efforts have largely focused on monoclonal antibodies directed against Aβ and hyperphosphorylated tau [7]. Simultaneously, glia-mediated neuroinflammation and metabolically driven disruptions of neural networks are increasingly recognized as key drivers of disease progression, offering novel avenues for therapeutic intervention. Moreover, growing evidence highlights the critical roles of the gut microbiota, epigenetic regulation, and neurogenesis in AD pathology. Strategies targeting these processes-including microbiota modulation, lifestyle interventions, small-molecule therapeutics, and natural compounds-hold promise for developing innovative approaches to AD treatment.

Based on these developments, this review synthesizes the core molecular mechanisms underlying AD and their complex interactions and provides a comprehensive overview of emerging targeted therapeutic strategies. We aim to provide an integrative framework that advances mechanistic understanding of AD and current target-based therapeutic strategies, and inform the development of next-generation disease-modifying interventions.

## The Key molecular pathways in Alzheimer's disease pathogenesis

**Fig. 1:**
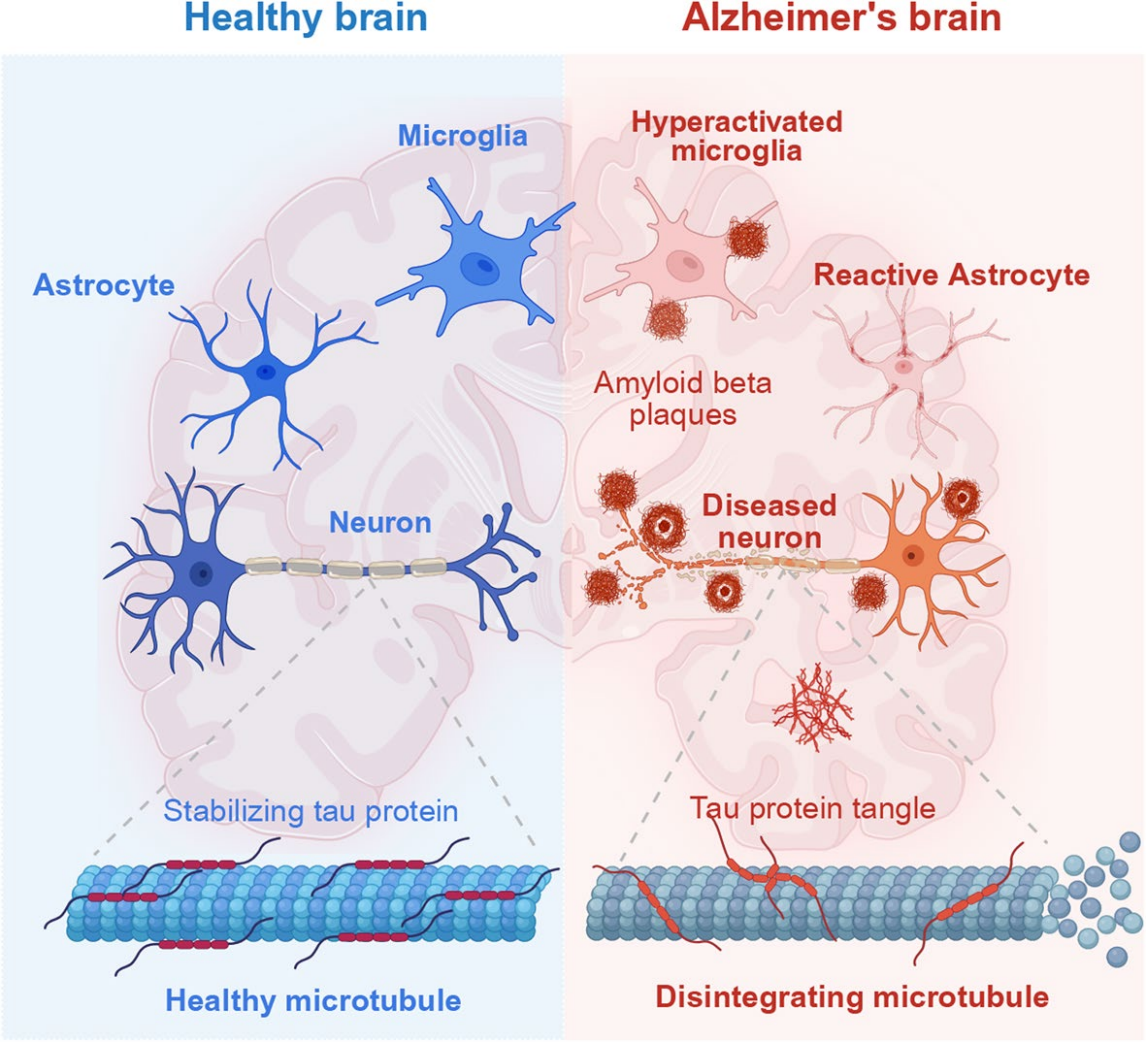
Key pathological alterations in the AD brain. Under physiological conditions, microglia and astrocytes provide neuroprotection. As the brain's primary resident immune cells, microglia modulate neuroinflammation by releasing cytokines and phagocytosing neuronal debris and pathological aggregates. Astrocytes, meanwhile, offer metabolic and structural support to neurons. Simultaneously, tau , as a soluble microtubule-associated protein, binds tubulin to assemble and stabilize microtubule structures, ensuring the normal transport of nutrients and signaling molecules within neurons. However, compared with the healthy brain, the AD brain exhibits pronounced atrophy and accumulates the canonical pathological hallmarks: Aβ plaques appear in the brain parenchyma, disrupting synaptic neural signaling. Abnormal tau aggregate into NFTs, destabilizing microtubule structures. Microglia and astrocytes become hyperactivated, releasing excessive pro-inflammatory cytokines that trigger intense neuroinflammation. Collectively, these processes lead to neuronal injury and death, ultimately driving the progressive neurodegeneration characteristic of AD

The pathogenesis of AD reflects the interplay of genetic, environmental, and lifestyle factors. Scientific understanding has shifted from focusing on single pathological hallmarks toward a more integrated view of the disease’s multidimensional biological mechanisms. Key neuropathological features include abnormal extracellular accumulation of Aβ plaques, intraneuronal hyperphosphorylated tau forming NFTs (Fig. 1). Multiple hypotheses have been proposed to explain AD onset and progression, among which the Aβ and tau hypotheses remain predominant due to their broad relevance across the disease continuum.

### The amyloid cascade hypothesis

Extensive deposition of Aβ plaques is a defining pathological hallmark of AD, first described in 1984 [8]. Building on this observation, Hardy and Higgins proposed the amyloid cascade hypothesis (ACH), which posits that accumulation of Aβ within the brain parenchyma initiates a sequence of pathological events culminating in neurodegeneration and progressive cognitive decline [9]. Over the subsequent decades, advances in preclinical, clinical, imaging, and genetic research have refined—but consistently reinforced—the centrality of Aβ deposition within this framework [10]. As such, the ACH has profoundly shaped the development of both pharmacological and non-pharmacological therapeutic strategies targeting Aβ as a primary pathogenic driver in AD.

Evidence supporting the ACH comes from pathological studies showing Aβ plaques in multiple brain regions of patients with AD, whereas such deposits are absent in cognitively healthy individuals [11]. Furthermore, plaque presence and density correlate closely with cognitive impairment [12]. Genetic evidence reinforces this hypothesis: (1) in familial AD, mutations in *amyloid precursor protein (APP), presenilin 1 (PSEN1)*, and *PSEN2* increase Aβ production, elevate the Aβ42/Aβ40 ratio, and promote plaque formation; (2) in Down syndrome, triplication of the wild-type *APP* gene leads to early cerebral Aβ deposition, accompanied by microglial proliferation, neurofibrillary tangles, and cognitive deficits later in life [13]; and (3) *Apolipoprotein E (ApoE)*, encoded by the polymorphic *APOE* gene on chromosome 19, occurs as three major alleles (*ε2, ε3*, and *ε4*) that differ in protein conformation, receptor-binding affinity, and stability. Among them, the *ε4* allele confers the strongest genetic risk for AD, exhibiting a dose-dependent association with disease risk, age at onset, and Aβ neuropathology. Despite these findings, Aβ-centered ACH remains a primary research framework, however, its validity has been questioned because anti-Aβ therapies have shown limited clinical efficacy and the hypothesis does not account for observations that approximately 60% of individuals over 85 exhibit pathological Aβ accumulation without cognitive impairment [14, 15].

The formation of Aβ plaques is triggered by multiple factors, including excessive production, impaired clearance, and protein aggregation [16, 17], with an imbalance between production and clearance representing the primary driver of plaque accumulation [18–20]. Aβ is primarily generated through aberrant cleavage of APP, a type I transmembrane glycoprotein comprising a short intracellular C-terminal domain, an Aβ region, and a large extracellular N-terminal domain [21]. Under physiological conditions, APP is cleaved by α-secretase, which recognizes the protein’s spatial conformation and distance within the membrane, cutting within the Aβ region at amino acids 15–17. This intrapeptide cleavage disrupts the full-length Aβ sequence, generating a soluble α fragment (sAPPα) and leaving a membrane-bound C-terminal α fragment (αCTF). Because the resulting fragments do not contain the intact Aβ sequence and sAPPα exhibits neuroprotective properties, this pathway prevents Aβ formation and participates in synaptic regulation [22]. Consequently, this processing route does not lead to amyloid deposition, is referred to as the non-amyloidogenic pathway [23], and constitutes the primary physiological metabolic route of APP in vivo.

In the amyloidogenic pathway, APP is first cleaved at its N-terminal domain by β-site APP cleaving enzyme (BACE), releasing soluble APP β (sAPPβ) and the membrane-bound β C-terminal fragment (βCTF). The βCTF is subsequently processed by γ-secretase at multiple sites, generating Aβ peptides of varying lengths, with Aβ40 being the most abundant species in the brain, while Aβ42 is more prone to aggregation and represents the key pathogenic form [24]. These cleavages occur at the termini of the Aβ sequence, releasing the intact C-terminal Aβ peptide. The full-length Aβ is inherently “sticky,” readily forming oligomers that can ultimately aggregate into extracellular plaques [16, 17]. Aβ plaques disrupt neuronal signaling, trigger inflammatory responses, and promote cerebral amyloid angiopathy, culminating in neuronal death and cognitive decline [25, 26]. Thus, the amyloidogenic processing of APP constitutes a central pathogenic pathway in AD. Under physiological conditions, Aβ production is balanced by enzymatic degradation. However, in AD, key Aβ-degrading proteases, including insulin-degrading enzyme, angiotensin-converting enzyme, and neprilysin are downregulated [27], leading to excessive accumulation of Aβ.

### Tau pathology and neurofibrillary tangles

Another hallmark pathological feature of AD is NFTs, composed of abnormally aggregated tau, most found in the somatodendritic compartments of neurons [28–30]. Tau was first identified in 1975 as a highly expressed, soluble microtubule-associated protein in central nervous system (CNS) neurons [30, 31]. Its amino acid sequence includes a positively charged microtubule-binding region (MTBR) that facilitates assembly with tubulin to form stable and mature microtubules [32]. In healthy neurons, microtubules not only provide structural support [33] but also constitute a major component of the cytoskeleton, maintaining cellular morphology and mediating the transport of nutrients and organelles. Adjacent to the MTBR is a proline-rich region (PRR), which is also positively charged under physiological conditions and is repelled by MTBR4 [34].

Aberrant post-translational modification (PTM) of tau is central to both its loss of normal function and acquisition of toxicity. In the early stages of AD pathology, the PRR undergoes PTMs such as phosphorylation, acetylation, and ubiquitination, which impart a negative charge to the PRR [34–36]. This charge alteration promotes electrostatic attraction with the positively charged MTBR, inducing conformational changes in tau that facilitate self-association and co-assembly into semi-soluble pre-tangle structures, including paired helical filaments (PHFs) and straight filaments (SFs), distributed throughout the somatodendritic compartment [37]. These filaments further aggregate into insoluble, silver-staining NFTs. Accumulation of NFTs reduces acetylated α-tubulin [35, 36, 38, 39], a key marker of microtubule stability. Consequently, abnormal tau aggregation disrupts the cytoskeleton, paralyzes intracellular transport, deprives synapses of essential nutrients and signaling molecules, and ultimately drives neurodegeneration [40, 41].

Among tau’s PTMs, phosphorylation has been the most extensively studied [42, 43]. Tau can be phosphorylated at multiple sites by a variety of kinases, including protein kinase A, protein kinase C, cyclin-dependent kinase-5 (CDK5), Ca^2^⁺/calmodulin-dependent kinase II, glycogen synthase kinase-3β (GSK-3β), and mitogen-activated protein kinases (MAPKs) [44, 45]. In the normal adult brain, each mole of tau contains 2–3 mol of phosphate; in AD brains, tau phosphorylation is increased two- to threefold [46]. Hyperphosphorylation of tau results from dysregulation of Ser/Thr kinases such as CDK5 and GSK-3β [45], with GSK-3 functioning as a key tau kinase and its activity critically modulated by the Wnt signaling pathway [47]. Hyperphosphorylated tau compromises axonal microtubule integrity and increases cellular susceptibility to oxidative stress by impairing peroxisome trafficking, ultimately leading to neuronal and synaptic dysfunction [48].

In the early stages of tau pathology, soluble phosphorylated tau (pTau) exists in a dynamic equilibrium and can be dephosphorylated by phosphatases such as calcineurin, restoring its normal function [49, 50]. This dephosphorylation occurs extremely rapidly postmortem, within approximately 15 min [51, 52], rendering early soluble pTau nearly undetectable in routine autopsy brain tissue and leading to its historical underappreciation [49, 50]. Soluble hyperphosphorylated tau can aggregate on microtubules and disrupt endosomal transport, exerting toxicity on dendritic integrity and neuronal function [53]. Furthermore, upon dissociation from microtubules, exposed MTBR domains of tau can interact with Low-Density Lipoprotein Receptor-Related Protein 1 (LRP1) on the cell membrane, facilitating the “seeding” of pTau between neurons and propagating tau pathology across excitatory neuronal networks. Accordingly, the earliest soluble pTau represents the most toxic and seeding-competent species [53–56] and constitutes a key driver of neuronal dysfunction [57].

Tau aggregates may exhibit prion-like properties, enabling their propagation throughout the brain [58]. Specifically, misfolded tau within a neuron can be released extracellularly and taken up by neighboring neurons, where it acts as a “seed” to induce misfolding and aggregation of endogenous tau. This process allows tau pathology to spread in an infection-like manner along neuronal connectivity pathways, following a predictable and stereotyped pattern (Braak stages I–VI), beginning in the entorhinal cortex, progressing to the hippocampus, and eventually affecting the entire neocortex [59]. This propagation closely mirrors the progressive cognitive decline observed in AD patients, providing a clear anatomical correlation of disease progression [60, 61]. While traditional views consider the formation of insoluble tau fibrils, such as NFTs, as the key toxic event in AD, studies confirm that NFTs density correlates with cognitive impairment [28, 29]. However, accumulating evidence indicates that smaller, soluble, non-fibrillar tau oligomers, referred to as “invisible tau” [62], play a more critical role in neurotoxicity and the propagation of tau pathology in the CNS [62–66].

### The interplay between Aβ and tau

Aβ and tau are linked by a potent positive feedback loop, with several key mechanistic nodes. First, tau mediates Aβ toxicity. Tau is an essential component of the Aβ-driven excitotoxicity pathway [67]. In multiple animal models, partial tau reduction protects Aβ-overexpressing AD mice from cognitive impairment, whereas Fyn overexpression exacerbates these deficits [68, 69]. The tau–Fyn–NMDAR signaling axis represents a central mechanism by which tau enables Aβ neurotoxicity [67, 70]. In addition, soluble pTau enhances Aβ production. Immuno-EM studies in aged nonhuman primates show that early soluble pTau species (e.g., pT217-tau) accumulate on dendritic microtubules and “trap” APP-containing endosomes [71], leading to APP retention in BACE-rich compartments and markedly increasing Aβ42 generation [72]. Second, Aβ oligomers amplify tau pathology and calcium dysregulation. Recent evidence demonstrates that Aβ mediates more than 70% of the association between extracellular matrix proteins such as *APOE* and tau pathology [73]. Aβ potentiates tau toxicity through two principal mechanisms: accelerating tau phosphorylation and promoting tau oligomerization. Aggregated Aβ increases the activity of GSK-3β [74] and CDK5 [75], and may activate MAPKs and GSK-3β to drive PHF-tau formation in AD brain [76, 77]. Moreover, Aβ enhances tau oligomerization through activated CDK5, GSK-3β, and caspase-3, facilitating the formation of soluble toxic tau oligomers and promoting their transcellular spread [78–81].

## Key contributing pathways and pathogenic cofactors

**Fig. 2:**
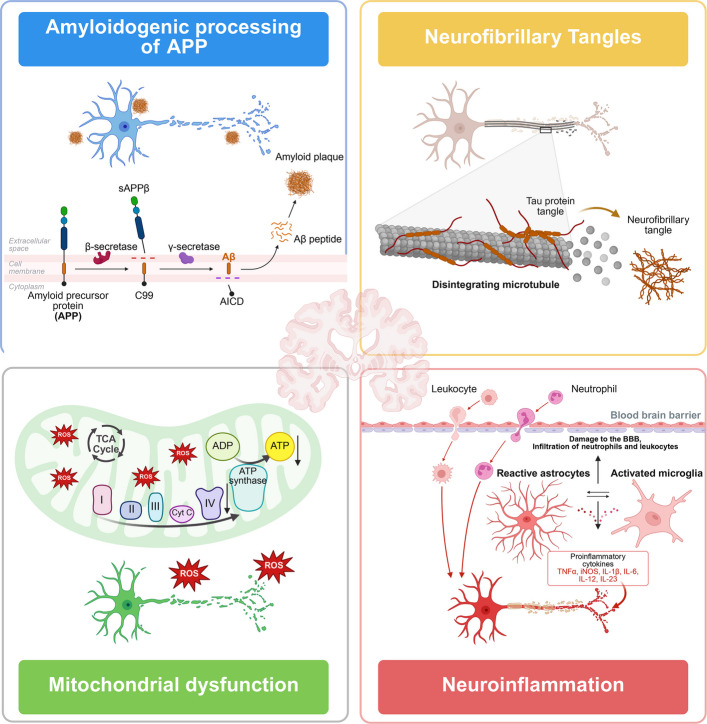
Schematic overview of mechanisms driving neuronal injury in Alzheimer’s disease. Progressive neuronal dysfunction and loss are central to cognitive decline in AD. Under pathological conditions, APP is sequentially cleaved by β-secretase and γ-secretase, generating neurotoxic Aβ peptides that aggregate into extracellular plaques and disrupt synaptic signaling. Tau undergoes abnormal phosphorylation and conformational changes, leading to the formation of NFTs. These aggregates destabilize microtubules, promote cytoskeletal disintegration, and impair axonal transport of nutrients and signaling molecules, ultimately resulting in synaptic dysfunction. Mitochondrial dysfunction represents another key mechanism in AD; oxidative stress and related insults disrupt oxidative phosphorylation in the electron transport chain, leading to neuronal energy deficits and increased vulnerability to degeneration. In addition, hyperactivated microglia and astrocytes accumulate around Aβ and NFTs, releasing pro-inflammatory mediators—including cytokines (e.g., TNF-α and IL-1β) and chemokines—and recruiting peripheral immune cells, thereby amplifying neuroinflammation and aberrant synaptic pruning. Collectively, these processes interact and reinforce one another, forming a pathogenic cascade that ultimately culminates in neuronal death and loss

The onset and progression of AD arise from the convergence of multiple pathogenic pathways and cooperating cofactors. Beyond the core lesions of Aβ deposition and tau hyperphosphorylation, disturbances in calcium homeostasis, mitochondrial dysfunction, synaptic impairment, neuroinflammation, lipid metabolic dysregulation, and genetic risk factors all contribute to a cascading disease process. These pathways are tightly interconnected and reinforce one another through multilayered positive-feedback mechanisms that drive neuronal injury and network deterioration (Fig. 2). A deeper understanding of these pathogenic components and their interactions helps explain the clinical and biological heterogeneity of AD and provides a foundation for subsequent discussions on mechanisms, biomarkers, and therapeutic strategies.

### Neuroinflammation

Accumulating evidence has highlighted neuroinflammation as another central driver of AD, rather than being merely a downstream consequence of AD pathology [82–87]. Neuroinflammation represents a complex cellular response of the brain to injury, trauma, or infection, acting as a double-edged sword: it can be protective to neurons under certain conditions but, when inflammatory signaling is overactivated, can damage brain cells [88, 89]. Key mediators include activated and proliferating microglia and astrocytes [90], which under normal conditions provide neuroprotection but become highly deleterious when pathologically overactivated. In AD brains, these cells cluster around NFTs and senile plaques, releasing cytokines, interleukins, and chemokines that amplify neuroinflammation and accelerate disease progression [91, 92].

Microglia are the principal resident immune cells in the brain and exhibit two major activation phenotypes, M1 and M2. The M1 phenotype is proinflammatory and neurotoxic, whereas the M2 phenotype contributes to resolution of inflammation and exerts neuroprotective effects. M1-polarized microglia upregulate proinflammatory cell-surface markers such as MHC-II and CD86 [93, 94] and produce a broad range of inflammatory mediators, including TNF, IL-1, IL-6, IL-12, IL-17, IL-18, and IL-23, thereby driving the initiation and progression of neuroinflammation [95–98]. In contrast, M2-polarized microglia release anti-inflammatory factors and neurotrophic molecules that promote immune resolution and tissue repair, mitigating neurotoxic inflammatory responses and supporting neuroprotection [99–101].

Under physiological conditions, microglia regulate neuroinflammation by releasing cytokines and by phagocytosing neuronal debris and pathological aggregates, thereby maintaining cerebral homeostasis [102–107]. In AD, microglia are tightly linked to both tau and Aβ pathology. In the early disease stage, microglia exhibit protective functions: they recognize and attempt to phagocytose Aβ plaques, and they actively participate in forming plaques composed of Aβ fibrils and associated proteins [108–110], promoting the development of insoluble, dense-core plaques [109]. Through these actions, microglia help maintain a dynamic balance between the generation and clearance of small, diffusible Aβ aggregates during the initial stages of AD [109]. At late stages, however, excessive accumulation of misfolded proteins and sustained microglial overactivation trigger robust neuroinflammation [111, 112]. Aberrant Aβ aggregation may initiate this inflammatory cascade, as soluble Aβ oligomers can activate and drive the proliferation of microglia [113, 114]. The resulting expansion of activated microglia leads to increased release of proinflammatory cytokines, which further promotes Aβ plaque formation and establishes a self-amplifying cycle that exerts direct neurotoxic effects-a phase often described as the destructive stage of microglial activation [115, 116]. In addition, tau aggregates and NFTs potentiate microglial activation and senescence, impairing their phagocytic capacity [117, 118]. Internalized tau can activate the NLRP3 inflammasome within microglia, facilitating tau seeding and accelerating AD-related pathology [87, 119].

In addition, Keren-Shaul and colleagues first identified disease-associated microglia (DAM) in AD mouse models in 2017 using single-cell transcriptomics [120–122]. DAM represents the terminal state along a continuum from homeostatic microglia to a disease-specific phenotype (stage II DAM) [123]. This phenotype is characterized by downregulation of homeostatic genes and upregulation of late-onset AD (LOAD) risk genes [123–125]. DAM exhibits a dual capacity to enhance both phagocytosis and inflammatory responses [126]. They show increased survival, migration, and chemotaxis, preferential clustering around Aβ deposits, elevated production of proinflammatory cytokines (IFN-γ, IL-1β, IL-6, TNF-α), and strengthened phagocytic activity driven by upregulation of lysosomal and phagosome-associated genes [123, 124, 127, 128]. DAM also displays substantial heterogeneity, comprising proinflammatory and anti-inflammatory subpopulations. Proinflammatory DAM emerges early and correlates positively with neuropathology, whereas anti-inflammatory DAM become more prominent in later disease stages [129].

Astrocytes in the brain also respond to immune cues. Under physiological conditions, their primary functions are to provide metabolic and structural support to neurons. Evidence from postmortem studies of individuals with mild cognitive impairment (MCI) or preclinical AD [130], as well as from animal models, indicates that astrogliosis may precede the formation of Aβ. Reactive astrocytes exhibit marked heterogeneity, including the neurotoxic A1 phenotype and the neuroprotective A2 phenotype [131]. A1 astrocytes are induced by activated microglia through proinflammatory cytokines [132]. Similar to microglia, astrocytes adopt a disease-associated state characterized by elevated glial fibrillary acidic protein (GFAP) and increased expression of genes linked to inflammatory signaling and toxic responses [133]. Astrocytes undergo molecular, functional, and morphological remodeling in response to AD pathology [132, 134]. They migrate toward Aβ plaques in response to monocyte chemoattractant protein-1 (MCP-1) and attempt to degrade them. Astrocytes also secrete glypican-4, which promotes tau hyperphosphorylation and thereby directly exacerbates tau pathology. Conversely, NFTs activate complement component 3 (C3) on astrocytes, further amplifying neuroinflammation and neuronal injury. Astrocytes additionally act in concert with microglia to propagate inflammatory signaling.

### Mitochondrial dysfunction

Neurons exhibit exceptionally high energy demands to sustain synaptic activity and plasticity, with mitochondria serving as the primary organelles responsible for ATP production [135]. Longitudinal studies indicate that mitochondrial dysfunction represents an early event in AD pathogenesis and may precede Aβ accumulation and tau pathology [136]. Widespread mitochondrial impairments have been documented in AD, including deficits in energy metabolism, heightened oxidative stress, disrupted mitochondrial quality control (biogenesis, dynamics, and mitophagy), and impaired calcium homeostasis [137]. The electron transport chain (ETC), a central component of oxidative phosphorylation, comprises complexes I–IV embedded in the inner mitochondrial membrane [138]. These complexes transfer electrons derived from glucose and fatty acids to molecular oxygen while pumping protons across the membrane to generate the electrochemical gradient required for ATP synthesis [139, 140]. In AD, mitochondrial dysfunction reduces ETC efficiency and diminishes ATP production, leading to severe energy deficits [141–143]. The resulting energy failure compromises neuronal viability and exacerbates cellular injury [144].

In AD, downregulation of PGC-1α impairs transcriptional activation of nuclear respiratory factors (NRFs) and mitochondrial transcription factor A (TFAM), resulting in insufficient mitochondrial DNA (mtDNA) replication and inadequate synthesis of essential mitochondrial proteins [145]. This deficiency compromises the oxidative phosphorylation (OXPHOS) system, reduces ATP production, and amplifies ROS generation, exacerbating mitochondrial dysfunction [145]. Mitochondrial dynamics involve two opposing GTPase-regulated processes—fusion and fission—that are critical for maintaining organelle function and adapting to cellular stress [146, 147]. In the prefrontal cortex of patients with AD, levels of dynamin-related protein 1 (DRP1) and fission protein 1 (Fis1) increase with disease progression [148]. Excessive DRP1 activation drives pathological mitochondrial fragmentation, impairs mitochondrial trafficking along axons and dendrites, and disrupts synaptic function [149].

Mitophagy is a selective degradation pathway essential for cellular homeostasis [150]. This process is primarily mediated by the PINK1/Parkin pathway, which is markedly disrupted at multiple steps—from initiation to lysosomal degradation—in AD. At the initiation stage, PINK1 mRNA is reduced in AD brain tissue [151, 152]. Hyperphosphorylated tau further distorts autophagy receptor structure and competes with light chain 3 (LC3) binding, thereby impairing the early recruitment machinery and blocking mitophagy initiation [153]. During autophagosome formation and trafficking, Aβ is enriched at mitochondria-associated membranes [154]. After entering mitochondria and localizing to the cristae, Aβ binds Aβ-binding alcohol dehydrogenase (ABAD) to enhance ROS generation and trigger neuronal apoptosis [155]. Aβ also interacts with Beclin-1 to impair autophagosome nucleation [156] and inhibits ATG4-mediated LC3 processing, disrupting mitochondrial recognition and sequestration [157]. Tau pathology further impairs autophagosome transport by binding to motor proteins and reducing their motility in a concentration-dependent manner [158]. At the lysosomal degradation stage, mitophagy strongly depends on lysosomal integrity, which is profoundly compromised in AD brains, thereby exacerbating mitochondrial dysfunction [159]. Downregulation of the lysosomal biogenesis regulator transcription factor EB (TFEB), together with reduced expression of lysosomal markers such as  lysosomal-associated membrane protein 1 (LAMP1) in AD models, further impairs degradative capacity [160]. Accumulation of Aβ within autophagic vesicles disrupts their fusion with lysosomes, amplifying mitochondrial defects [160]. In addition, tau sequesters Parkin in the cytosol and prevents its recruitment to damaged mitochondria, thereby weakening mitophagic clearance [161].

### Metabolic dysregulation

Metabolic dysregulation is a fundamental pathological feature of AD, characterized primarily by impaired glucose metabolism, insulin resistance, and disrupted lipid homeostasis. Reductions in cerebral glucose metabolism represent an early diagnostic hallmark of AD [162–164]. These deficits are driven by two major processes: impaired glucose transport across the blood–brain barrier (BBB) and defects in intracellular glucose catabolism, the latter being partly dependent on mitochondrial function [165]. Cerebral glucose utilization is tightly regulated by glucose Transporters (GLUTs) to ensure adequate delivery to neurons and glial cells for energy production. In AD, reduced O-GlcNAcylation suppresses the expression of GLUT1 and GLUT3 [166]. The resulting oxidative stress perturbs cellular membranes and disrupts signaling pathways that regulate transporter expression, establishing a self-reinforcing cycle of progressive bioenergetic failure [167, 168]. Moreover, Aβ plaques can markedly impair cellular glucose uptake [169], and reduced glucose availability subsequently limits substrate entry into the tricarboxylic acid cycle, diminishing the generation of NADH and FADH₂ [170, 171].

Insulin resistance in the brain also contributes to the pathogenesis of AD. Cerebral insulin resistance is observed in AD patients, characterized by increased serine phosphorylation of insulin receptor substrate-1 (IRS-1) in the hippocampus and cortex, which correlates with tau and Aβ pathology [172]. Impaired insulin signaling reduces AKT phosphorylation, resulting in dysregulated GSK-3β activity, which promotes tau hyperphosphorylation and NFTs formation, exacerbating ATP depletion and oxidative stress [173, 174]. Insulin resistance also disrupts signaling via the mTORC1 and mTORC2 complexes, which regulate metabolic activity, mitochondrial biogenesis, autophagy, and protein synthesis [173]. Impaired mTOR activity further compromises mitophagy, leading to the accumulation of dysfunctional mitochondria [173]. Meanwhile, chronic neuroinflammation can aggravate insulin resistance, as pro-inflammatory cytokines, including tumor necrosis factor-α and interleukin-6, impair insulin signaling and promote oxidative stress [175].

Lipid metabolism dysregulation represents another pathogenic mechanism in AD. Lipids are abundant in the CNS and play critical roles in cellular structure, signal transduction, and energy homeostasis [176, 177]. AD is characterized by widespread alterations in blood lipids and regional brain lipid composition, including fatty acids and broader lipid metabolic pathways [178–181]. For instance, elevated serum low-density lipoprotein cholesterol has been associated with AD [182, 183], while monoacylglycerols and diacylglycerols increase in the frontal cortex and plasma during early disease stages [184], and plasma triglycerides are reduced in MCI and AD [185]. Most cholesteryl esters, particularly long-chain species, decreased in AD [186, 187]. Cholesteryl esters, as storage forms of excess cholesterol, have been identified as upstream regulators of tau pathology, and their reduction enhances proteasome activity, lowering tau phosphorylation [188]. Recent studies show that overexpression of the low-density lipoprotein receptor (LDLR) significantly reduces *APOE* levels and mitigates neurofibrillary degeneration and neurodegeneration in P301S mice [189–191].

### Synaptic dysfunction and neurotransmitter deficits

Analyses of AD brains have shown functional impairment and selective loss of multiple proteins essential for maintaining synaptic structure and function, including synapsin, synaptophysin, and neurogranins. The reduction of these proteins is closely correlated with the severity of dementia [192, 193]. In the hippocampus, early AD is marked by decreased levels of key postsynaptic proteins involved in synaptic morphology and plasticity, including drebrin and postsynaptic density protein 95(PSD-95) [194]. Beyond synaptic proteins, microRNAs (miRNAs) have gained increasing attention in recent years [195–197]. miRNAs are small non-coding RNAs that regulate gene expression [198, 199] and contribute to synaptic dysfunction in AD by modulating the expression of synaptic proteins and transcription factors [195, 196, 200–203]. They exert distinct functions across synapse formation, maturation, plasticity, transmission, and termination [196]. For example, miR-455-3p regulates genes involved in APP processing and Aβ production and influences mitochondrial and synaptic biogenesis pathways [200, 204, 205]. Dysregulation of miR-455-3p disrupts these pathways and contributes to synaptic loss, impaired neurotransmission, and cognitive deficits characteristic of AD [204]. Similarly, miR-128 reduces tau phosphorylation and Aβ accumulation by inhibiting key regulatory proteins such as GSK-3β, Aβ precursor protein binding protein 2 (APPBP2), and mammalian Target of Rapamycin (mTOR) [206].

Selective neuronal populations undergo degeneration in AD, leading to profound disruptions in key neurotransmitter systems. The cholinergic system-comprising neurons that produce acetylcholine-is essential for memory, learning, and cognition [207]. A marked reduction in ACh is a central contributor to memory impairment and cognitive decline in patients with AD. In AD, acetylcholinesterase (AChE) activity is significantly increased, accelerating the degradation of synaptic ACh, reducing its availability, and thereby diminishing the efficiency of neuronal signaling. The nucleus basalis of Meynert in the basal forebrain is the principal source of cholinergic projections to the cerebral cortex. Neurons within this region undergo substantial degeneration, resulting in impaired cholinergic regulation, reduced cholinergic innervation of the cortex and hippocampus, and a pronounced decline in ACh levels [208], collectively contributing to deficits in memory and attention [209].

### Genetic risk factors

AD is highly heritable, with an estimated 60–80% of disease risk attributable to genetic factors. Its genetic architecture is complex, involving contributions from rare, high-penetrance variants as well as common alleles of modest effect. For instance, APOE, a secreted protein produced primarily by astrocytes, plays central roles in lipid transport, modulation of neuroinflammation, and neuronal repair [210–213]. A large study of 28,864 participants demonstrated that ε4 carriers represent a substantial proportion of late-onset AD cases [214], and individuals with ε4 show higher Aβ plaque burden and earlier Aβ pathology [215, 216]. APOE regulates Aβ aggregation and clearance through direct interactions that depend heavily on its lipidation state; poorly lipidated APOE is prone to oligomerization, forming scaffolds that facilitate Aβ aggregation, while its reduced affinity for Aβ impairs microglial-mediated clearance [217–219]. Beyond Aβ-dependent mechanisms, APOE also exacerbates tau propagation and tau-associated neurodegeneration. Notably, deletion of endogenous *APOE* mitigates neurodegeneration without altering tau protein levels, suggesting that its effects are mediated largely through downstream processes such as microglial-driven neuroinflammation [220, 221]. APOE isoforms further modulate the aggregation of both Aβ and tau and directly shape microglial inflammatory responses in AD [220, 222].

Advances in modern genomics, particularly large-scale genome-wide association studies, have identified more than 70 AD-associated genetic loci, underscoring the marked heterogeneity and polygenic nature of the disease [223–226]. Among these, *TREM2 *represents a key risk gene whose function is primarily mediated by microglia within the CNS, highlighting the tight intersection between genetic susceptibility and immune responses at the microglial level [227]. *BIN1*, another major risk factor for sporadic AD, contributes to disease pathogenesis by regulating L-type voltage-gated calcium channels and thereby driving calcium dysregulation [228], and may also facilitate tau internalization and propagation [229].

## Emerging pathways: beyond classical pathogenic mechanisms

An increasing number of emerging pathogenic pathways have been shown to play critical roles in the onset and progression of AD. For example, gut microbiota dysbiosis can influence the CNS through the gut-brain axis. Epigenetic alterations, meanwhile, bridge genetic susceptibility and environmental exposures, exerting long-lasting regulation over genes involved in synaptic function, inflammatory responses, and protein homeostasis, thereby contributing to disease progression. Impaired neurogenesis is closely associated with declines in learning and memory. These emerging pathways intertwine with and amplify classical pathological mechanisms, collectively forming a complex and dynamic pathogenic network that offers new avenues for early diagnosis and targeted intervention.

### Gut microbiota

The gut microbiota has emerged as one of the most notable pathogenic mechanisms in AD research in recent years. Metagenomic analysis of fecal samples across five stages of pathological progression in AD patients revealed that over 10% of microbial species undergo significant changes during disease development [230]. AD is characterized by systemic and intestinal inflammation, accompanied by a decrease in the abundance of *Firmicutes* and an increase in *Bacteroidetes* [231]. Specific gut microbial features have also been linked to preclinical AD, with pathways such as arginine and ornithine degradation showing a correlation coefficient as high as 0.967 with preclinical AD status [232]. Preclinical studies have demonstrated a causal relationship between the gut microbiota and AD pathology through microbial transplantation experiments. For example, fecal samples from AD patients transplanted into Thy1-C/EBPβ transgenic mice pretreated with a broad-spectrum antibiotic cocktail induced AD-like pathology, accompanied by microglial activation and cognitive deficits, with *Bacteroides fragilis* identified as a key pathogenic species [233]. Transplantation of gut microbiota from patients into healthy young adult rats also induced typical AD-associated cognitive impairments and reduced neurogenesis [231]. Similarly, fecal microbiota transfer from AD patients exacerbated neuropathology and cognitive deficits in AD mouse models [234, 235]. Oral exposure to *Escherichia coli (E. coli)* exacerbates cognitive deficits and increases Aβ pathology in the 5xFAD mouse model of AD [236], underscoring the critical role of the gut microbiota in AD pathogenesis.

At the molecular level, the gut microbiota exerts its effect primarily through microbial metabolites. For instance, indole-3-propionic acid (IPA) levels in feces and serum are significantly reduced in individuals with MCI and AD, and serum IPA correlates strongly with cognition. IPA crosses the BBB and activates neuronal pregnane X receptor (PXR), inhibiting NF-κB activation and BACE1 expression, thereby reducing Aβ accumulation and ameliorating cognitive deficits in AD mice [237]. Dysbiosis can also lead to peripheral accumulation of phenylalanine and isoleucine, which stimulate proinflammatory Th1 cell proliferation and differentiation, subsequently activating M1 microglia and promoting AD progression [238]. Furthermore, microbiota secretes substantial amounts of lipopolysaccharide (LPS), which may enhance microglial activation and proinflammatory cytokine production in the AD brain, while other microbial metabolites such as short-chain fatty acids (SCFAs) and trimethylamine N-oxide (TMAO) contribute to AD pathology through mechanisms including neuroinflammation, neurotransmitter imbalance, and mitochondrial dysfunction [239]. In addition, the gut serves as a significant source of peripheral Aβ, which can be transported to the brain via the circulation, and induces neuroinflammation in APP/PS1 mice, recapitulating phenotypes characteristic of early AD pathology [240].

### Epigenetic regulation

Epigenetic modifications, including DNA methylation, chromatin remodeling, histone modifications, and noncoding RNA regulation-modulate gene expression without altering the underlying DNA sequence, thereby integrating external stimuli into the genome [241]. Analysis of the epigenomic and transcriptomic landscapes of 850,000 nuclei from the prefrontal cortex of 92 individuals with and without AD revealed widespread epigenomic dysregulation in the brains of late-stage AD patients [242]. Genome-wide DNA methylation profiling using methylation arrays identified significant methylation changes in lipid-regulatory genes such as* CTNNB1* and *SLC27A1*, as well as in the lysosomal transmembrane gene *TMEM175*, suggesting that abnormal epigenetic regulation of lysosomal genes and disrupted lipid homeostasis represent important risk factors for AD [243]. Histone lysine lactylation (Kla) represents a novel epigenetic modification. H3K18la is markedly upregulated in senescent microglia, as well as in the hippocampi of naturally aged and AD model mice. Its elevated expression directly activates the NF-κB signaling pathway, promoting the senescence-associated secretory phenotype and thereby accelerating both aging and AD-related phenotypes [244]. In a separate study, histone lactylation was found to be increased in brain tissue from 5xFAD mice and AD patients, with H4K12la specifically enriched in microglia adjacent to Aβ plaques. This lactate-dependent histone is concentrated at the promoters of glycolytic genes, enhancing transcription and glycolytic activity, and ultimately exacerbating microglial dysfunction in AD [245].

A recent study identified reduced lactylation at APP K612 as a critical pathological alteration driving amyloidogenic processing of APP in AD brain tissue. The APP K612T mutation, by enhancing the interaction between APP and CD2-associated protein (CD2AP) within endosomes, promotes APP degradation via the endosome-lysosome pathway [246]. In Drosophila neurons, overexpression of Aβ induces aberrant histone acetylation (H3K9ac) in glial cells and completely disrupts presynaptic homeostasis at neuromuscular junctions [247]. Succinylation, a metabolism-linked PTM, was quantitatively profiled in the brains of AD patients and healthy controls, revealing pronounced increases at key lysine residues of APP and microtubule-associated tau. Succinylation of APP disrupts its normal proteolytic processing, thereby promoting Aβ accumulation and plaque formation, whereas tau succinylation facilitates its aggregation into NFTs and impairs microtubule assembly [248]. Therefore, epigenetic regulation has also been recognized as a potential therapeutic target for AD [249, 250].

### Neurogenesis

The hippocampus is a central hub for cognition and memory and is among the earliest brain regions affected in patients with AD. The dentate gyrus (DG), a subregion of the hippocampus, is closely associated with learning and memory, particularly pattern separation. A distinctive feature of the DG is that it harbors a so-called “neurogenic niche”, in which stem cells continuously generate new neurons in the adult brain through a specialized form of cellular plasticity known as adult hippocampal neurogenesis (AHN) [251]. Adult-born hippocampal neurons synaptically integrate into mammalian hippocampal circuits and contribute to structural and functional plasticity, thereby supporting cognitive functions such as spatial learning, pattern separation, and memory, as well as emotional regulation [252]. Accumulating evidence indicates that patients with AD exhibit a marked reduction in neurogenesis [253], and with disease progression, both the number and the degree of maturation of newly generated neurons decline progressively [254]. These findings suggest that impaired neurogenesis may represent an important potential mechanism underlying memory deficit in AD and other neurodegenerative diseases [255].

Single-nucleus RNA sequencing has revealed a reduction in the number of immature granule cells (imGCs) in patients with AD, accompanied by profound alterations in gene expression profiles. As imGCs are critical for maintaining synaptic plasticity, their loss is likely to be closely associated with the decline in memory and cognition observed in AD [256]. In 5xFAD mice, impairment of hippocampal neurogenesis precedes the onset of behavioral deficits, and activation of adult-born neurons in the hypothalamus ameliorates memory and affective impairments in AD model mice [257]. Moreover, enhancing neurogenesis restores the number of newly recruited neurons within memory engrams, rescues dendritic spine density, and reshapes the transcriptional profiles of both immature and mature neurons, ultimately leading to the recovery of contextual and spatial memory [258]. In both postmortem brain tissue from patients with AD and AD mouse models, astrocytes exhibit upregulated expression of chitinase-3-like protein 1 (CHI3L1), which impairs neurogenesis via the CRTH2-IKKβ-S6K1 signaling pathway. Targeting its receptor, CRTH2, to inhibit CHI3L1 effectively restores neurogenesis and cognition in AD mice [259]. Collectively, these findings indicate that disrupted neurogenesis may represent a key pathogenic mechanism underlying memory impairment in AD, and that targeting hippocampal neurogenesis or developing pro-neurogenic therapies holds promise as a potential therapeutic strategy for AD [260].

## From pathways to targets: evolution of therapeutic strategies

**Fig. 3:**
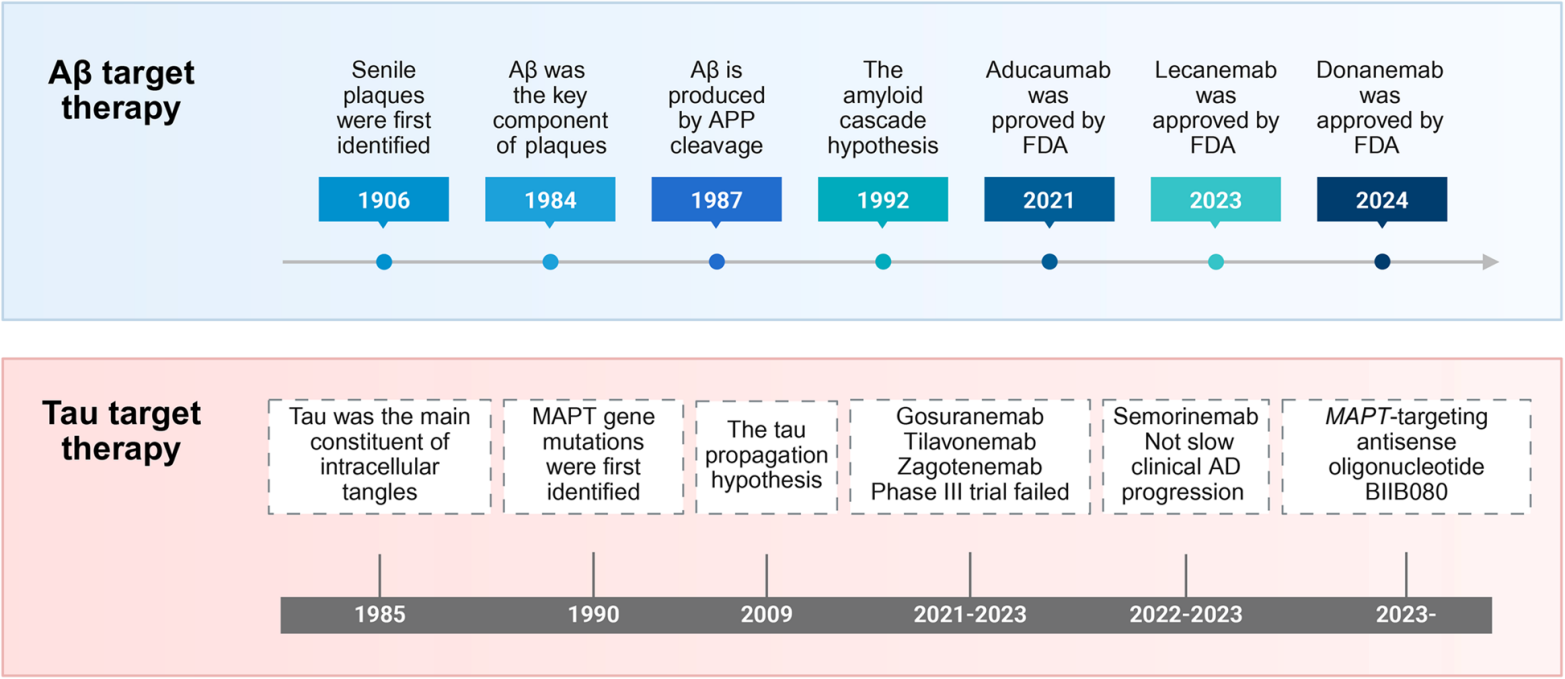
Milestones in the development of Aβ-targeted and tau-targeted therapies. This figure summarizes key milestones in the discovery of Aβ and tau pathologies, the identification of associated genes, the formulation of major pathogenic hypotheses, and the development of targeted therapeutics. After decades of drug development, the anti-Aβ monoclonal antibodies Aducanumab (2021), Lecanemab (2023), and Donanemab (2024) received FDA approval, representing significant advances in disease-modifying therapies. In contrast, between 2021 and 2023, several tau-targeting immunotherapies (including Gosuranemab, Tilavonemab, Zagotenemab, and Semorinemab) failed to meet primary endpoints in clinical trials. Meanwhile, the antisense oligonucleotide BIIB080, which reduces tau translation by targeting MAPT pre-mRNA and promoting its degradation, has demonstrated promising therapeutic potential in clinical studies

In recent years, key pathological nodes, including Aβ deposition, tau hyperphosphorylation, neuroinflammation, and metabolic disruption, have been progressively delineated. Therapeutic concepts in AD have shifted from broad pathway modulation toward precise targeting of core pathogenic processes. In this section, we highlight recent advances in therapeutics targeting Aβ, tau, inflammatory signaling, and metabolic dysfunction, and outline emerging strategies and candidate agents that may inform next-generation interventions. We summarized the key historical milestones in Aβ- and tau-targeted therapeutic research in Fig. 3.

### Anti-Aβ therapies

**Fig. 4:**
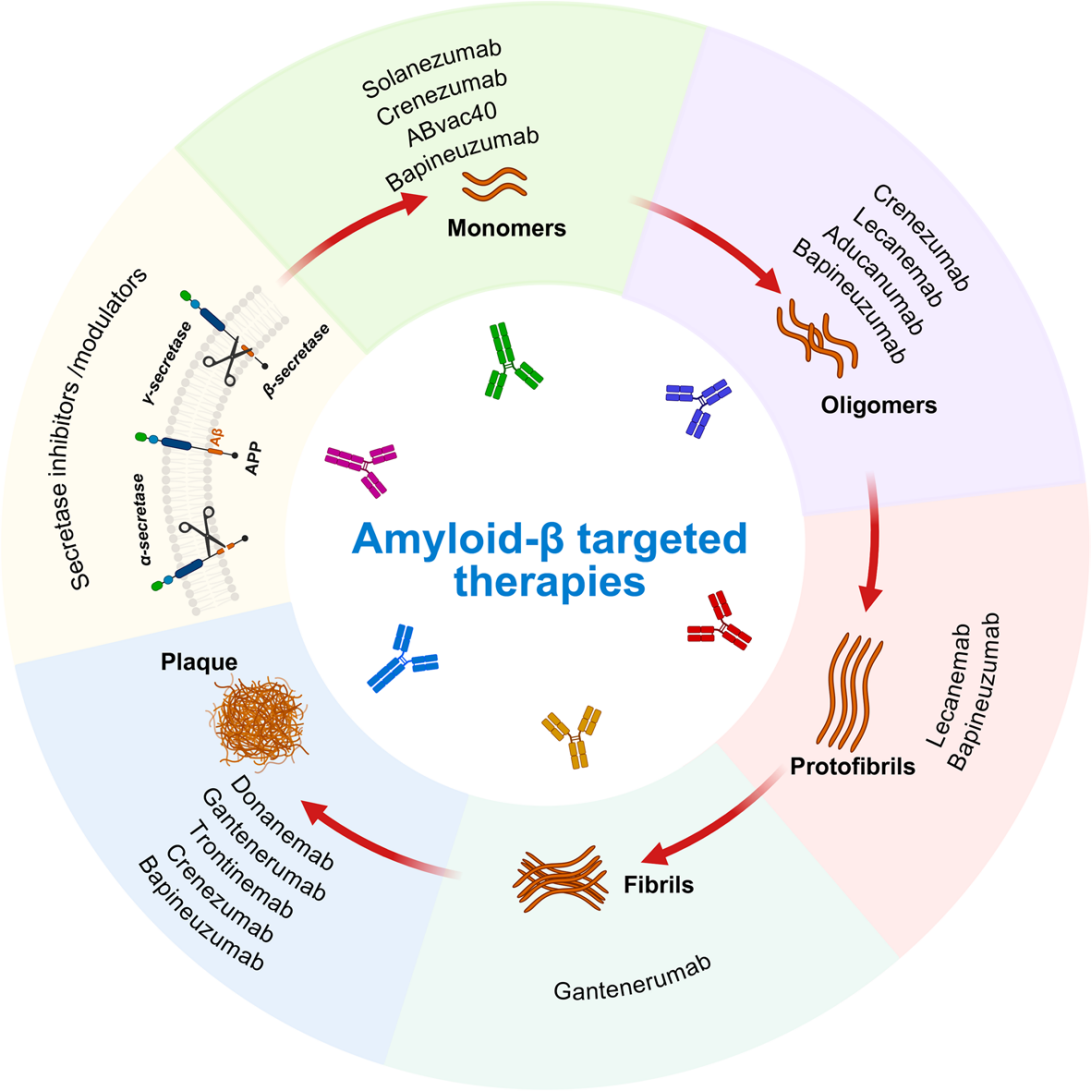
Illustration of Aβ-targeted therapies. This figure summarizes representative therapeutic agents targeting Aβ in AD. Aβ arising from sequential cleavage of APP by β- and γ-secretases, in contrast to the non-amyloidogenic α-secretase pathway. Aβ monomers aggregate into oligomers, protofibrils, and fibrils, ultimately forming neurotoxic amyloid plaques. Therapeutic strategies include secretase inhibitors/modulators that reduce Aβ production and immunotherapies that enhance Aβ clearance by targeting distinct conformational and aggregation states. Monomer-targeting antibodies include Solanezumab, ABvac40, Crenezumab, and Bapineuzumab. Antibodies against oligomers or soluble aggregates include Aducanumab, Crenezumab, Bapineuzumab, and Lecanemab, with Bapineuzumab and Lecanemab also binding protofibrils. Gantenerumab primarily targets fibrillar Aβ. Antibodies recognizing N-terminally truncated or modified Aβ species in plaques include Donanemab, Gantenerumab, Crenezumab, Bapineuzumab, and Trontinemab

Therapeutic strategies targeting Aβ have primarily focused on reducing its production via secretase inhibitors or enhancing its clearance through immunotherapy [14] (Fig. 4). Additionally, recent studies have highlighted the potential of agents that inhibit Aβ aggregation [261]. Table 1 summarizes recent studies on Aβ-targeted therapeutic strategies and their principal findings.

**Table 1:** A summary of recent studies on Aβ-targeted therapeutic strategies and their principal findings

Drug	Mechanism of action	Study population	Phase	Conclusion	Trial ID	Ref
**Bapineuzumab**	Monoclonal antibody specific to the N-terminus of the Aβ protein designed to decrease plaque formation and promote clearance of Aβ	Mild to moderate AD	phase 3	No effect of bapineuzumab on amyloid load or cerebrospinal fluid phosphorylated tau	NCT00667810 NCT00676143	[262]
**Solanezumab**	Monoclonal antibodies for monomeric amyloid proteins	Mild dementia of AD/Dominantly inherited AD	phase 3	Did not significantly affect cognitive decline	NCT01900665NCT01760005	[263, 264]
**Aducanumab**	Preferentially recognizes aggregated forms of Aβ and facilitates plaque removal	Mild cognitive impairment or mild dementia of AD	phase 3	In EMERGE, the high-dose group showed a statistically significant slowing of clinical decline on the primary endpoint (CDR-SB) and on three secondary endpoints (MMSE, ADAS-Cog13, and ADCS-ADL-MCI)	NCT02484547 NCT02477800	[265]
**Lecanemab**	Monoclonal antibodies targeting protofibrils and plaques, preferentially targets Aβ protofib	Mild cognitive impairment or mild dementia of AD	phase 3	Reduced markers of amyloid in early AD and resulted in moderately less decline on measures of cognition and function	NCT03887455	[266]
**Donanemab**	An immunoglobulin G1 monoclonal antibody directed against insoluble, modified, N-terminal truncated form of Aβ present only in brain Aβ plaques	Early AD	phase 2/3/3b	Donanemab improved composite measures of cognition and daily functioning and most effectively slowed tau accumulation in participants with complete amyloid clearance and in brain regions affected later in the pathological sequence. At 76 weeks, it significantly delayed clinical progression in individuals with low to medium tau levels, as well as in the combined population with low/medium and high tau pathology	NCT03367403NCT04437511NCT05108922NCT05738486	[267–271]
**Gantenerumab**	A subcutaneously administered, fully human, anti-Aβ IgG1 monoclonal antibody with highest affinity for aggregated Aβ, including oligomers, fibrils, and plaques	Dominantly inherited AD	phase 2/3	Gantenerumab markedly reduced amyloid plaques, CSF tTau and pTau181, and attenuated increases in NfL. It also decreased CSF neurogranin and plasma GFAP while increasing CSF sTREM2, but showed limited effects on cognition. Partial or short-term Aβ clearance produced no significant clinical benefit	NCT01760005NCT06424236NCT04623242	[264, 272, 273]
		Mild cognitive impairment or mild dementia of AD	phase 3	Gantenerumab significantly reduced amyloid plaque burden and modulated cerebrospinal fluid and plasma biomarkers. It was associated with an increased rate of whole-brain volume loss, while hippocampal volume remained unchanged	NCT03444870 NCT03443973	[274–276]
**Crenezumab**	A humanized anti-Aβ monoclonal immunoglobulin G4 antibody, binds monomeric and aggregated Aβ, with higher affinity for oligomeric Aβ	Prodromal to mild (early) AD	phase 3	Crenezumab was well tolerated but did not reduce clinical decline in participants with early AD	NCT02670083 NCT03114657	[277]
**ALZ-801/valiltramiprosate**	A small-molecule oral inhibitor of Aβ aggregation and oligomer formation	APOE4 carriers with early AD	phase 2	ALZ-801 lowered plasma p-tau181 over two years, attenuated hippocampal atrophy, and was not associated with vasogenic cerebral edema	NCT04693520	[278]
**Sabirnetug (ACU193)**	A humanized monoclonal antibody selective for soluble Aβ oligomers	mild cognitive impairment and mild dementa of AD	phase 1	After three doses, sabirnetug showed biomarker changes suggesting potential neuroprotective effects	NCT06335173	[279]
**Solanezumab**	An immunoglobulin G1 monoclonal antibody that binds to the mid-domain of the Aβ monomer	Preclinical AD	phase 3	Did not slow cognitive decline as compared with placebo over a period of 240 weeks in persons with preclinical AD	NCT02008357	[280]
**KHK6640**	A novel humanized anti-Aβ oligomer-specific antibody	Mild to moderate AD	Phase 1/2a	KHK6640 was well-tolerated across all doses, without any amyloid-related imaging abnormalities for edema, and amyloid-related imaging abnormalities for hemorrhage was as population background	/	[281]
**Posiphen**	An orally administered small molecule, binds to an iron-responsive element in APP mRNA and decreases translation of APP and Aβ	Early AD	phase 1b	Posiphen was safe and well-tolerated in Early AD	NCT02925650	[282]

This list of preclinical projects is not exhaustive but illustrates representative recent studies evaluating Aβ-targeted therapeutic agents

*Aβ* amyloid-β, *AD* Alzheimer's disease, *ARIA-E* Amyloid-related imaging abnormalities Edema, *CDR-SB* Clinical Dementia Rating-Sum of Boxes, *CSF* cerebrospinal fluid, *GFAP* glial fibrillary acidic protein, *sTREM2* soluble triggering receptor expressed on myeloid cells 2

Direct infusion of monoclonal antibodies to rapidly clear Aβ represents the most advanced and currently the only approved disease-modifying therapy, ushering in a new era of AD treatment [283]. First-generation monoclonal antibodies, which targeted non-toxic Aβ monomers-including bapineuzumab (fibrillar Aβ), solanezumab (NCT02008357, monomeric Aβ), and crenezumab-demonstrated modest reductions in brain Aβ burden but failed to produce cognitive benefits in phase III clinical trials [284–286]. The turning point in Aβ immunotherapy came with second-generation antibodies, which are directed against pathogenic Aβ species and aggregates. Representative agents include aducanumab, lecanemab, donanemab, and gantenerumab [287]. Aducanumab (NCT04241068), a human monoclonal antibody selectively targeting aggregated Aβ, penetrates the brain in AD transgenic mouse models, binds parenchymal Aβ, and dose-dependently reduces both soluble and insoluble Aβ. Preclinical studies with murine chimeric aducanumab demonstrated effective plaque clearance but did not attenuate microglial proliferation; instead, it exacerbated astrocytic activation associated with plaques, suggesting potential activation of cytotoxic pathways and other adverse consequences [288]. In patients with prodromal or mild AD, monthly intravenous infusion of aducanumab for 12 months reduces brain Aβ levels and improves cognitive performance [289], with Aβ-related imaging abnormalities-edema (ARIA-E) being the most common adverse event [290]. Although aducanumab received accelerated FDA approval in 2021, its clinical efficacy remains highly controversial [291].

Lecanemab, a humanized IgG1 monoclonal antibody with high affinity for soluble Aβ protofibrils, reduces Aβ-related biomarkers in early AD and slows cognitive and functional decline over 18 months compared with placebo [266]. Lecanemab (NCT03887455) received full FDA approval in 2023, though safety concerns persist [292, 293]. A recent real-world study in China reported clinical efficacy of lecanemab in patients with AD-MCI and mild AD, while outcomes were less favorable in more advanced cases, supporting its favorable safety profile in clinical practice [294]. Donanemab is an IgG1 monoclonal antibody targeting an insoluble, N-terminally truncated, post-translationally modified form of Aβ confined to amyloid plaques. In a phase 3 trial, donanemab slowed AD progression [269]. Gantenerumab, a fully human, subcutaneously administered anti-Aβ IgG1 antibody with high affinity for aggregated Aβ, including oligomers and fibrils, showed limited efficacy in early symptomatic AD [274]. In the four-year DIAN-TU-001 trial, gantenerumab produced no improvement in neurodegenerative imaging biomarkers or neuropathology [295].

These antibodies underscore the therapeutic potential of Aβ targeting but share a risk of ARIA and primarily slow, rather than halt, disease progression [296]. Additional Aβ-directed antibodies are under investigation. Sabirnetug (ACU193) selectively targets soluble Aβ oligomers and crosses the BBB, engaging its target in early-phase studies (INTERCEPT-AD, NCT04931459) [279]. ABBV-916 binds N-terminally truncated, pyroglutamate-modified Aβ (AβpE3) and effectively reduces plaques in preclinical models without inducing microhemorrhages, supporting its potential as a next-generation therapeutic currently in clinical evaluation [297].

### Therapeutic strategies targeting tau

**Fig. 5:**
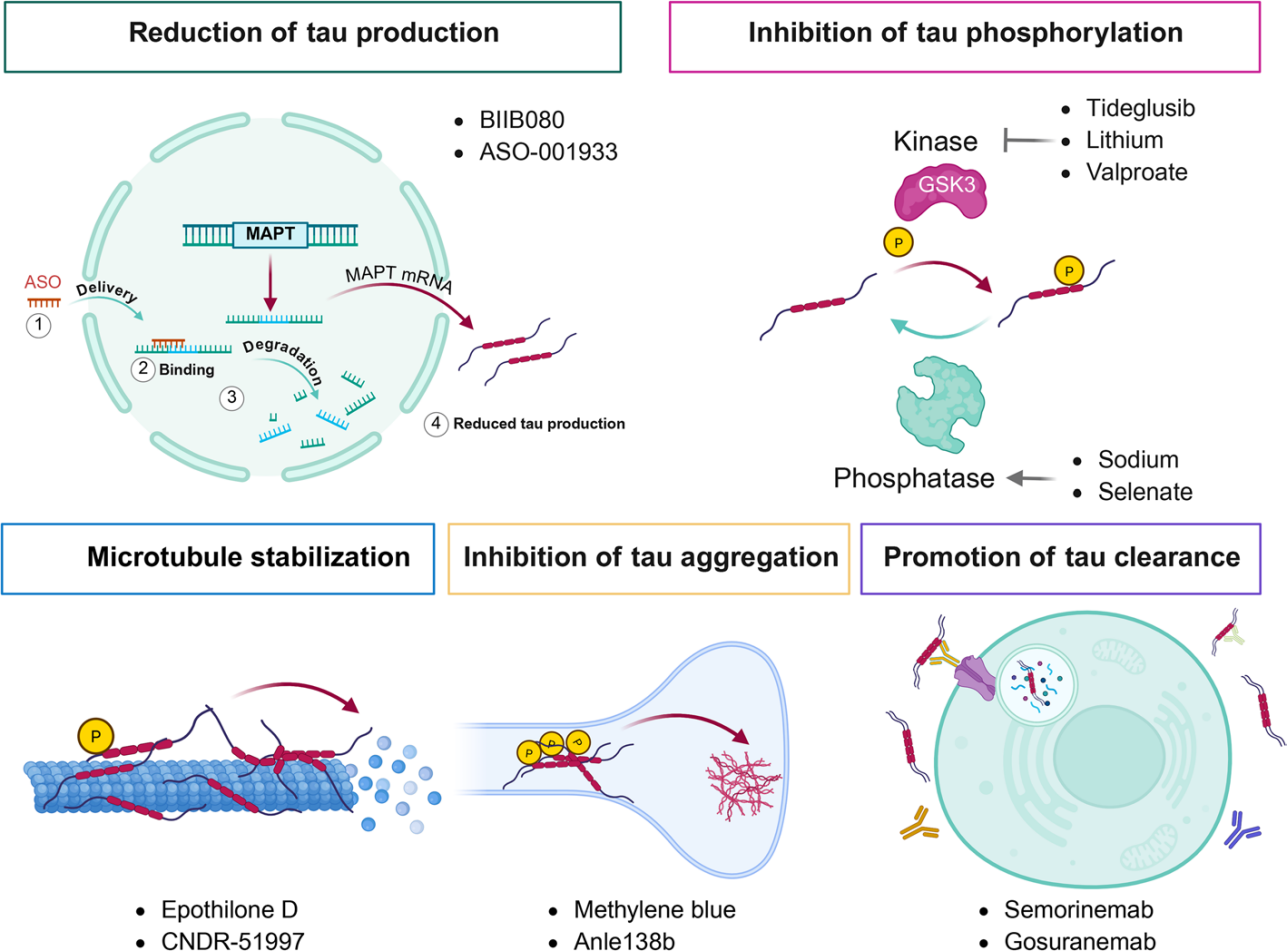
Schematic diagram of intervention strategies targeting tau pathology. Therapeutic strategies targeting tau pathology aim to reduce tau production, inhibit hyperphosphorylation, stabilize microtubules, prevent aggregation, and enhance clearance. Antisense oligonucleotides (BIIB080, ASO-001933) degrade MAPT pre-mRNA to suppress tau translation. Hyperphosphorylation is limited via kinase inhibitors (Tideglusib, Lithium, Valproate) or phosphatase activators (sodium selenate). Microtubule-stabilizing agents (Epothilone D, CNDR-51997) preserve cytoskeletal integrity. Small-molecule aggregation inhibitors (Methylene blue, Anle138b) prevent or reverse tau aggregation. Immunotherapies (Semorinemab, Gosuranemab) promote immune-mediated clearance by targeting specific tau domains, particularly the N-terminus

In recent years, tau-targeted approaches-including immunotherapy, small molecules, antioxidant enzymes, and gene therapy-have entered clinical development. These therapies aim to inhibit tau hyperphosphorylation, prevent tau aggregation, promote tau clearance, or reduce tau production (Fig. 5). Table 2 summarizes recent studies on tau-targeted therapeutic strategies and their principal findings. Strategies targeting tau hyperphosphorylation largely focus on GSK-3 as a primary target. Tideglusib, a GSK-3β inhibitor, significantly reduced APP and pTau in the hippocampus of AD mice and improved spatial learning and memory. However, a phase II trial in patients with mild-to-moderate AD (NCT01350362) failed to meet its primary endpoints [304]. Computational screening of Tideglusib-based multi-target analogs recently identified SG-09 as a potential GSK-3β inhibitor [305]. With emerging evidence for GSK-3α’s role in AD pathology, selective inhibition of GSK-3α has been proposed; for example, an ATP-competitive GSK-3α inhibitor studied by Brenda Amaral et al. reduced tau phosphorylation in neonatal rat brains, suggesting potential to slow early pathology [306].

**Table 2:** A summary of recent studies on tau-targeted therapeutic strategies and their principal findings

Drug	Mechanism of action	Study population	Phase	Conclusion	Trial ID	Ref
**MAPT**_**Rx**_** (ISIS 814907/BIIB080)**	An antisense oligonucleotide designed to reduce concentrations of MAPT messenger RNA	Mild AD	phase 1b	MAPT_Rx_ was well tolerated and resulted in a sustained reduction of tau protein levels. Reduced tau biomarkers, including CSF t-tau, CSF p-tau181, and tau PET, which is associated with cognitive decline, in participants with mild AD	NCT03186989	[298, 299]
**Semorinemab**	A humanized IgG4 monoclonal antibody that targets the N-terminal domain of tau	Prodromal to mild AD	phase 2	Semorinemab did not slow clinical AD progression compared with placebo throughout the 73-week study period but did demonstrate an acceptable and well-tolerated safety profile	NCT03289143	[300]
		Mild to moderate AD	phase 2	Semorinemab was safe and well tolerated, did not slow functional decline in patients with mild-to-moderate AD	NCT03828747	[301]
**ACI-35.030** **JACI-35.054**	A SupraAntigen®-based liposome A CRM197 carrier-protein conjugate	Early AD	phase 1b/2a	The two active immunotherapies were well tolerated and generated robust titres against pathological Tau species	NCT04445831	[302]
**Etalanetug**	An anti-tau monoclonal antibody, is intended to inhibit spreading of pathologic tau species by binding to the microtubule binding region	Healthy volunteers	phase 1	Etalanetug had an adequate safety and immunogenicity profile in healthy adults	/	[303]

This list of preclinical projects is not exhaustive but illustrates representative recent studies evaluating tau-targeted therapeutic agents

*CSF* cerebrospinal fluid, *AD* Alzheimer's disease

A recent study demonstrated that lithium exerts anti-dementia effects by inhibiting GSK-3β, thereby reducing tau phosphorylation, and by modulating downstream GSK-3 signaling, including upregulation of BDNF and Bcl-2 and stabilization of disrupted calcium homeostasis, supporting brain health and long-term cognitive function [307]. Another strategy to reduce tau phosphorylation targets O-GlcNAcylation. O-GlcNAc modification at serine and threonine residues antagonizes phosphorylation, and O-GlcNAcase inhibitors increase tau O-GlcNAc levels to suppress multi-site hyperphosphorylation and fibrillization. Tau aggregation inhibitors primarily rely on small molecules designed to prevent misfolding and assembly or to disassemble existing  NFTs. Hydromethylthionine mesylate is a representative agent, acting to inhibit tau aggregation and depolymerize pathological tau oligomers and filaments [308, 309].

Tau immunotherapy is categorized into active (vaccines) and passive (monoclonal antibodies) approaches, both designed to harness the immune system to clear pathological tau. AADvac1 (NCT02579252) is the first tau vaccine to enter clinical trials and has been shown to delay AD-related cognitive decline via antibody-dependent mechanisms [310]. In a preclinical study using a Qβ virus-like particle vaccine targeting pTau at threonine 181, the pT181-Qβ vaccine effectively reduced tau phosphorylation, insoluble tau burden, and NFTs in PS19 and hTau mouse models as well as in young adult non-human primates, highlighting its therapeutic potential [310]. Passive immunotherapy involves monoclonal antibodies targeting distinct pathological tau epitopes. Tilavonemab (NCT02880956), an IgG4 antibody binding the N-terminus of human tau and soluble extracellular tau, failed to slow disease progression or improve cognition in early AD patients [311]. Etalanetug (E2814) is a monoclonal antibody that targets the MTBR of tau to inhibit pathological tau propagation [303]. Semorinemab, a humanized IgG4 antibody recognizing the N-terminal domain (residues 6–23) and all full-length tau isoforms, did not reduce brain tau accumulation or slow clinical decline in a phase II AD trial (NCT03289143) [300, 301].

### Anti-neuroinflammation therapies

Considering the critical contribution of central neuroinflammation and peripheral immune responses to the pathogenesis of AD, targeting inflammatory pathways has emerged as a new therapeutic direction [312]. Current anti-inflammatory strategies primarily focus on modulating microglia and astrocytes as key cellular mediators of neuroinflammation (Table 3).

**Table 3:** A summary of recent therapeutic strategies targeting novel AD–related pathways and their principal findings

Drug	Mechanism of action	Study population	Phase	Conclusion	Trial ID	Ref
LM11A-31	Functions as a p75NTR modulator to downregulate its degenerative signaling and as an antagonist to pro-NGF-induced degeneration	Mild to moderate AD	phase 2a	LM11A-31 was generally well tolerated in a population with mild to moderate AD	NCT03069014	[313]
AL101 (GSK4527226)	A monoclonal antibody therapy in development for AD that targets sortilin to elevate functional PGRN levels	Healthy volunteers	phase 1	Both single and multiple doses of AL101 led to significant increases in plasma and CSF PGRN levels	NCT04111666	[314]
CT1812	An orally bioavailable, brain penetrant small molecule antagonist of the sigma‐2 receptor complex to block Aβ oligomer toxicity	Mild to moderate AD	phase 1b/2a	CSF Aβ oligomers increased with CT1812, indicating displacement and clearance of toxic oligomers	NCT02907567	[315]
Septin glue REM0046127	It acts by normalizing cytosolic Ca^2^⁺ levels without disrupting the physiological functions of Ca^2^⁺	Mild‐to‐moderate AD	phase 2a	Showed liver adverse effects	NCT05478031	[316]
Intranasal insulin and empagliflozin	Improve insulin sensitivity and vascular function	MCI or early AD	phase 2a/b	INI and empagliflozin treatment was safe with promising effects on cognition, fluid, and imaging biomarkers	NCT05081219	[317]
Masitinib	An orally administered tyrosine kinase inhibitor that targets activated cells of the neuroimmune system	Mild-to-moderate AD	phase 3	Masitinib (4.5 mg/kg/day) may benefit people with mild-to-moderate AD	NCT01872598	[318]
AL002	An investigational, engineered, humanized monoclonal immunoglobulin G1 antibody designed to target TREM2	Healthy volunteers	phase 1	Single-dose AL002 showed central nervous system penetrance and was well tolerated, with no treatment-related serious adverse events over 12 weeks	NCT03635047	[319]
Deferiprone	An orally bioavailable iron chelator that has superior access to the brain	MCI or early AD	phase 2	Deferiprone 15 mg/kg twice a day decreased hippocampal QSM and accelerated cognitive decline in patients with amyloid-confirmed early AD	NCT03234686	[320]
low-dose Interleukin-2	Expanded Treg populations, suppressed monocytes inflammatory markers, and reduced plasma inflammatory cytokines	mild to moderate AD	phase 2a	The IL-2 immunotherapeutic strategy was safe and well-tolerated. IL-2 q4wks effectively leading to modification in inflammatory mediators and CSF Aβ42 levels	NCT06096090	[321]
Intermittent senolytic combination therapy of dasatinib plus quercetin	Clear senescent cells	Early AD	preclinical	/	NCT04063124	[322, 323]
Semaglutide	Improve neuroinflammatory pathways, vascular and BBB integrity, reduced synaptic loss, and neuroprotection	Early AD	phase 3	/	NCT04777396 NCT04777409	[298]

The list of preclinical programs is not meant to be exhaustive, but rather to illustrate the extent of effort underway and the different target modalities being deployed

*AD* Alzheimer's disease, *PGRN* Progranulin, *CSF* cerebrospinal fluid, *Aβ* amyloid-β, *TREM2* Triggering Receptor Expressed on Myeloid Cells 2, *MCI* mild cognitive impairment, *QSM* Quantitative Susceptibility Mapping, *BBB* Blood–Brain Barrier

Modulators targeting microglia represent a major branch of neuroinflammation-directed therapy and include TREM2 agonists, TLR4 inhibitors, and tyrosine kinase inhibitors. A representative agent, AL002 (NCT04592874), is a humanized agonistic monoclonal antibody against TREM2. Preclinical studies show that TREM2 activation enhances microglial uptake and clearance of Aβ, whereas loss-of-function variants increase the risk of sporadic AD, supporting TREM2 as an attractive therapeutic target [324]. In crab-eating macaques, weekly intravenous AL002 administration for 4 weeks was well tolerated and produced dose-dependent reductions in soluble TREM2 (sTREM2) in CSF, accompanied by decreases in total TREM2 in the hippocampus and frontal cortex and increases in biomarkers of TREM2 pathway activation in CSF and brain tissue. In a phase 1 study involving 64 healthy volunteers, a single intravenous infusion of AL002 demonstrated central target engagement, evidenced by dose-dependent reductions in CSF sTREM2 and corresponding increases in TREM2-pathway biomarkers and indices of microglial recruitment [319]. However, in the phase 2 INVOKE-2 trial (NCT04592874), AL002 did not significantly slow clinical progression in patients with AD [325]. To improve the efficacy of TREM2-targeted interventions, future trial designs may need to better account for AD heterogeneity and the dynamic activation states of microglia. Incorporating stratification based on TREM2 genotype, baseline sTREM2 levels, and transcriptional or functional markers of microglial state may help identify patient subgroups most likely to benefit from treatment [325]. In a preclinical study, researchers designed R@AClipo, a nanotherapeutic platform that co-delivers the TREM2 agonist peptide COG1410 and the glutamate modulator riluzole via Angiopep-2-modified liposomes capable of crossing the BBB. In an AD mouse model, R@AClipo upregulated TREM2 expression and enhanced microglia-mediated Aβ clearance [326].

Astrocyte-targeted modulators primarily focus on inhibiting key signaling pathways mediating neuroinflammation, such as NF-κB/NLRP3, JAK/STAT3, calcineurin/NFAT, and p38 MAPK. Various inhibitors targeting these pathways have shown promise in preclinical studies; for example, the STAT3 inhibitor Stattic, calcineurin inhibitor FK506, and p38 MAPK inhibitors MW181 and NJK14047 all demonstrated beneficial effects in animal models. Clinical-stage candidates include Pepinemab (NCT04381468), a humanized monoclonal antibody that neutralizes Semaphorin 4D‌ (SEMA4D), a molecule predominantly expressed in glial cells and critical for regulating the transition between glial homeostasis and reactive states. In a Phase II study evaluating Pepinemab for early Huntington’s disease, no significant therapeutic effects were observed, but the treatment was well tolerated, with a relatively low incidence of serious adverse events [327].

### Therapies for metabolic dysfunction

Given the multifactorial etiology of AD, metabolic dysfunction has emerged as a potential therapeutic target (Table 3). Insulin resistance plays a critical role in AD pathophysiology, making antidiabetic agents a potential strategy to target central insulin signaling [172, 328]. Intranasal insulin administration delivers insulin directly to the CNS, correcting glucose dysregulation, reducing neuroinflammation, and improving cognitive function in AD [329, 330]. Sodium–glucose cotransporter 2 inhibitors (SGLT2i) not only lower systemic glucose and sodium levels but may also enhance central insulin sensitivity and cerebrovascular function. A real-world study investigating glucagon-like peptide-1 (GLP-1) receptor agonists and SGLT2 inhibitors as potential AD therapies indicated that both drug classes may offer preventive or therapeutic benefits [331]. A recent Phase 2a/b randomized trial in patients with MCI and early AD evaluated intranasal insulin (INI) and the SGLT2i empagliflozin (EMPA). Both interventions were well tolerated; INI improved cognition, modulated MRI fractional anisotropy (FA) and cerebral blood flow (CBF), and reduced plasma GFAP levels. EMPA treatment lowered CSF tau and regulated CBF. Both drugs influenced multiple immune and inflammatory biomarkers, highlighting their potential as metabolic modulators in early AD [317].

Homeostatic regulation of metal ions is critical for maintaining normal nervous system function. Increasing evidence indicates that dysregulation of zinc, copper, calcium, and other metal ions not only affects intracellular redox balance and neuronal survival but is also closely associated with Aβ deposition, mitochondrial dysfunction, and cognitive decline. Consequently, metal ion dyshomeostasis has emerged as a novel focus in AD pathogenesis research and therapeutic strategy development. Zinc homeostasis genes primarily regulate intracellular and extracellular zinc concentrations, and their abnormal expression can induce neuronal apoptosis, oxidative stress, and neuroinflammation, ultimately promoting Aβ aggregation. Supplementation with zinc chelators has been proposed as a potential intervention for neurodegenerative diseases [332]. In AD patients, serum levels of free and total copper are significantly elevated, whereas brain copper content is reduced [333]. Copper can form complexes with Aβ, triggering oxidative stress, apoptosis, and cognitive deficits. In a recent clinical study using zinc therapy for patients with MCI (EudraCT No. 2019-000604-15), zinc treatment stabilized cognitive function in individuals with systemic copper imbalance, suggesting copper homeostasis as a potential early therapeutic target in AD [334]. Calcium dyshomeostasis contributes to mitochondrial dysfunction in AD, and restoring calcium balance is critical for improving mitochondrial health [335]. Brain lithium levels are significantly reduced in MCI patients, promoting Aβ deposition and tau hyperphosphorylation, accelerating cognitive decline. Alternative lithium therapies using salts with minimal Aβ binding potential may represent a promising preventive and therapeutic strategy for AD [336].

## Emerging therapeutic strategies: insights from preclinical evidence

As noted above, emerging pathogenic pathways play a critical role in the progression of AD. Strategies targeting these pathways-including modulation of the gut microbiota, lifestyle interventions, small-molecule drugs, natural compounds, and traditional Chinese medicines-have demonstrated therapeutic potential in preclinical studies and may offer novel avenues and directions for AD treatment.

### Gut microbiota–based strategies

Strategies aimed at modulating the gut microbiota may markedly restore intestinal homeostasis and exert beneficial effects on cognitive decline in numerous preclinical studies of AD [337]. Supplementation of *Bacteroides* and lysophosphatidylcholine (LPC) in AD model mice reduces Aβ plaque burden, restores synaptic function, improves cognitive deficits, attenuates gliosis, and mitigates myelin degeneration, suggesting that *Bacteroides ovatus* and LPC interventions hold promise as potential therapeutic approaches for AD [338]. *Akkermansia muciniphila*, a beneficial gut bacterium, is decreased in both the feces and blood of patients with AD. Administration of *A. muciniphila* before disease onset in AD mice improves mitochondrial fission, mitophagy, and cognition [339]. Furthermore, *A. muciniphila* treatment alleviates neuroinflammation and reduces Aβ deposition in APP/PS1 mice via modulation of the AhR/NF-κB/NLRP3 signaling pathway [340]. Oral supplementation with the *A. muciniphila* metabolite propionate maintains mitochondrial homeostasis in AD pathophysiology by downregulating the mitochondrial fission protein DRP1 through G protein-coupled receptor 41 (GPR41) and enhancing PINK1/PARKIN-mediated mitophagy via GPR43 [339]. *Bifidobacterium*, a key probiotic in the gut, plays a critical role in maintaining microbial homeostasis [341]. Oral administration of *Bifidobacterium pseudolongum* improves cognitive function and mitigates AD pathology. Its metabolite, propionic acid (PA), binds to FFAR3 to inhibit JNK phosphorylation and reduces NF-κB-regulated downstream neuroinflammatory factors, including TNF-α and IL-1β, thereby attenuating neuroinflammation [342]. This evidence underscores the potential of gut microbiota-targeted interventions as a promising strategy for modulating AD pathology and cognition decline.

### Lifestyle interventions

Lifestyle interventions, including dietary patterns and exercise, are increasingly recognized for their therapeutic potential in AD [343]. A randomized clinical trial demonstrated that adherence to a Western-style diet in cognitively normal middle-aged adults may increase AD risk by disrupting metabolic health, promoting AD-like pathology, reducing cerebral perfusion, and potentially impairing cognitive function. In contrast, a Mediterranean diet, characterized by high intake of fruits, vegetables, whole grains, fish, and olive oil, supports metabolic and brain health [344] and is associated with a reduced risk of cognitive impairment[345]. A low-carbohydrate modified Mediterranean ketogenic diet (MMKD) has been shown to improve peripheral lipid and glucose metabolism, including reductions in HbA1c, insulin, and triglyceride levels, while increasing cerebral ketone uptake [346].

Intermittent fasting (IF) is a dietary regimen characterized by alternating periods of eating and fasting within a defined temporal cycle. IF has been shown to improve cognitive function and ameliorate AD-like pathology in transgenic AD mouse models (5xFAD) via the gut microbiota-metabolite-brain axis. Specifically, IF significantly enriches beneficial bacteria such as *Lactobacillus*, reduces carbohydrate metabolism, and increases levels of creatine and dimethylglycine, collectively mitigating cognitive decline, lowering Aβ burden, and suppressing excessive glial activation in AD mice [347]. In APP/PS1 mice, IF also exerts therapeutic effects by upregulating pathways related to apoptosis, primary and secondary bile acid metabolism, and fatty acid biosynthesis, thereby effectively reducing Aβ deposition and cognitive impairment [237]. These findings suggest that IF may represent a potential strategy for preventing AD progression [347]. Similarly, MMKD significantly reshapes the gut microbiome and its metabolic outputs, promoting the growth of *Lactobacillus* and increasing bacterially derived lactate production. These serum metabolite changes upregulate specific neuroprotective receptors and induce alterations in neuroinflammation-related signaling pathways in the hippocampus [348].

Exercise also represents a key lifestyle-based intervention. Exercise training reduces circulating proinflammatory signaling molecules, protects against Aβ-mediated neuronal damage, and promotes AHN in AD rats [349]. High-intensity interval training alleviates cognitive impairment in streptozotocin -induced AD rat models by promoting the glymphatic drainage of Aβ from the cortex and hippocampus toward the kidneys [350]. Exercise also modulates the nuclear translocation of SUMO1 and IGF1R in the hippocampus, thereby facilitating neuronal regeneration while suppressing neuroinflammation, ultimately improving cognitive performance in APP/PS1 mice [351]. Furthermore, long-term physical activity may exert preventive effects against AD by downregulating the EAF2–p53–TSP-1 signaling pathway associated with reactive astrocytes, which enhances the plasticity and drainage capacity of meningeal lymphatic vessels [352].

### Small molecules

Fingolimod is an orally administered small-molecule immunomodulator capable of crossing the BBB, primarily acting through functional antagonism of sphingosine-1-phosphate (S1P) receptors. Administration of fingolimod during the larval stage rescues histone acetylation in glial cells and presynaptic homeostasis at neuromuscular junctions, and in Aβ-expressing Drosophila models, it improves adult neurodegeneration and motor function, highlighting the therapeutic potential of FTY720 in neurodegenerative diseases [247]. Histone deacetylase 6 (HDAC6) is a key member of the HDAC family, regulating multiple cellular functions including misfolded protein clearance and immune responses, and is closely associated with Aβ and tau pathology. The small-molecule HDAC6 inhibitor PB118 exhibits a multifaceted mechanism of action, clearing Aβ fragments via phagocytosis, increasing acetylated α-tubulin to stabilize microtubule networks, and markedly reducing tau phosphorylation and neuroinflammation, thereby alleviating AD-related neuropathology [353]. The ErbB4 receptor plays a critical role in AD pathogenesis. The small-molecule ErbB4 agonist E4A effectively activates ErbB4, upregulates DOCK3 and SIRT3 expression, and suppresses TLR4–NF-κB–NLRP3 pathway activation, thereby reducing neuroinflammation and Aβ plaque formation and improving cognitive deficits in APP/PS1 mice [354]. Survivin, a member of the inhibitor of apoptosis protein (IAP) family, promotes hippocampal neurogenesis when overexpressed and enhances cognitive function in 5xFAD mice, suggesting that survivin may represent a promising therapeutic target for AD [355]. Receptor tyrosine kinases (RTKs) can directly bind oligomeric Aβ, promoting its deposition, disrupting synaptic structure and plasticity in AD. Small-molecule inhibitors and monoclonal antibodies targeting RTKs have shown promising therapeutic potential in preclinical studies [356]. Thus, small-molecule interventions act on specific targets through diverse mechanisms, positioning them as promising therapeutic candidates for AD.

### Natural compounds and traditional Chinese medicines

Eucommiae cortex polysaccharides (EPs), among the most abundant constituents of the Eucommiae cortex, alleviate learning and memory deficits in AD models in a gut microbiota–dependent manner [357]. Pseudostellaria heterophylla polysaccharide (PH-PS) reshapes gut microbiota composition, restores intestinal barrier function, and reduces the secretion of proinflammatory cytokines to attenuate peripheral inflammation. It promotes the conversion of M1 microglia and A1 astrocytes toward neuroprotective M2 and A2 phenotypes, respectively, and facilitates Aβ plaque clearance by upregulating the expression of insulin-degrading enzyme and neprilysin [358]. Administration of Phyllanthus emblica polysaccharides (PEP) markedly mitigates cognitive decline in AlCl₃-treated rats. Mechanistically, PEP upregulates autophagy-related proteins (Atg5, Beclin1, LC3B) and LRP1 expression, while downregulating AD-related proteins, including BACE1, APP, Aβ, and p-Tau^Ser404, thereby ameliorating AD-like pathology [359]. Dietary capsaicin increases levels of 24(S)-hydroxycholesterol (24-HC) in the host, a change that correlates with an enrichment of the gut bacterium *Oscillibacter*. Elevated 24-HC enhances microglial phagocytic activity in the brain and suppresses proinflammatory cytokine production via liver X receptor β (LXRβ)-mediated transcriptional regulation [360].

Smilagenin, a lipid-soluble steroidal sapogenin extracted from the traditional Chinese medicinal herb Radix Asparagi, exerts neuroprotective effects in APP/PS1 mice by upregulating P300 expression. This increases histone acetylation at the BDNF promoter and enhances its transcription, effectively reducing Aβ plaque deposition and improving cognitive and learning abilities [361]. Curcumin administration enhances AHN in AD mice by targeting the PI3K/Akt pathway to regulate GSK-3β/Wnt/β-catenin and CREB/BDNF signaling, thereby improving cognition [362]. The traditional Chinese medicine formula Zexieyin increases the number of BrdU/DCX double-positive cells and Ki67-positive cells in the DG, promoting neurogenesis and ameliorating learning and memory deficits in AD mouse models [363]. Ginsenoside RK3, a rare ginsenoside derived from ginseng, promotes neurogenesis through activation of the CREB/BDNF pathway and improves learning and cognitive performance in APP/PS1 mice [364]. 20S-protopanaxatriol stimulates neural stem cell proliferation and neurogenesis via the PI3K/AKT pathway, thereby ameliorating cognitive deficits in AD models [365]. Collectively, these natural compounds and traditional herbal interventions demonstrate translational potential for AD therapy.

## Conclusions and prospects

This review provides a comprehensive summary of the core pathological features and classical pathogenic mechanisms of AD. It also highlights the emerging contributions of gut microbiota dysbiosis, aberrant epigenetic regulation, and impaired neurogenesis to AD progression. Based on this foundation, the review summarizes key recent developments in AD therapeutics, with a focus on clinical evidence from Aβ- and tau-targeted immunotherapies. It further outlines preclinical and clinical studies targeting neuroinflammation and metabolic dysfunction. Finally, emerging strategies for AD prevention and treatment are discussed, including gut microbiota modulation, lifestyle interventions, small-molecule drugs, natural products, and traditional Chinese medicine, along with their potential mechanisms of action. Collectively, these insights provide a valuable framework for optimizing therapeutic strategies and guiding the development of novel interventions for AD.

Despite significant recent advances, several challenges remain. First, therapies targeting specific pathological hallmarks, such as Aβ monoclonal antibodies, achieve precise intervention but have demonstrated only modest overall clinical efficacy. This suggests that relying on a single agent to substantially alter disease trajectory may be overly optimistic, reflecting the inherent complexity of AD pathogenesis and underscoring the need for multidimensional, mechanism-based interventions. Complementary strategies targeting different Aβ isoforms, tau aggregation, immune regulation, and gut microbiota may provide synergistic benefits, more effectively slowing or ameliorating disease progression. Second, the BBB, while critical for maintaining CNS homeostasis, significantly limits drug delivery to brain tissue. Integration of nanomedicine platforms and physical modulation strategies, such as focused ultrasound with microbubbles, holds promise for enhancing drug penetration and precision delivery, offering new avenues for targeted AD therapy.

Significant research progress has been made in recent years regarding biomarkers such as pTau181, pTau217, and pTau231. These markers are closely associated with the occurrence and progression trajectory of Aβ deposition and are now recognized as important molecular indicators reflecting pathological changes in AD. Additionally, biomarkers reflecting neuroinflammatory states, such as GFAP and TREM2, have emerged as research focal points, offering substantial clinical value for early AD diagnosis, disease monitoring, and treatment efficacy assessment. Integrating analyses of molecular and cellular changes reflected by these biomarkers will enable more comprehensive and dynamic evaluations of disease progression and treatment outcomes. Future research should focus on identifying biomarkers that capture early AD pathogenesis, thereby refining disease onset characterization and delineating its progression trajectory.

Finally, AD exhibits significant biological and clinical heterogeneity, which substantially influences individual patient responses to treatment and clinical outcomes. For instance, risk alleles such as *APOE ε4* are not only closely associated with Aβ deposition and disease progression rates but may also affect patient responsiveness to targeted therapies like anti-Aβ immunotherapy and the risk of adverse reactions. Concurrently, gender differences in neuroinflammatory responses, hormone levels, and brain structural changes may also contribute to treatment response variations. Furthermore, AD represents the combined outcome of multiple interacting pathological mechanisms, with different patients potentially exhibiting distinct disease subtypes dominated by Aβ pathology, tau pathology, neuroinflammation, or vascular factors. Future research requires larger longitudinal cohorts and clinical trials to systematically integrate genetics, humoral biomarkers, imaging features, and clinical information. This will identify key stratification markers predictive of treatment response and enable more precise patient classification and risk stratification.

## Data Availability

Not applicable.

## Abbreviations

AD: Alzheimer disease
Aβ: Amyloid β
NMDA: N-methyl-D-aspartate
NFTs: Neurofibrillary tangles
ACH: The amyloid cascade hypothesis
APP: Amyloid precursor protein
PSEN1: Presenilin 1
PSEN2: Presenilin 2
APOE: Apolipoprotein E
sAPPα: Soluble APPα
αCTF: α C-terminal fragment
BACE: β-Site APP cleaving enzyme
sAPPβ: Soluble APP β
βCTF: β C-terminal fragment
MTBR: Microtubule-binding region
PRR: Proline-rich region
PTMs: Post-translational modifications
CDK5: Cyclin-dependent kinase-5
GSK-3β: Glycogen synthase kinase-3β
pTau: Phosphorylated tau
LRP1: Low-density lipoprotein receptor-related protein 1
DAM: Disease-associated microglia
LOAD: Late-onset AD
MCI: Mild cognitive impairment
GFAP: Glial fibrillary acidic protein
ACh: Acetylcholine
AChE: Acetylcholinesterase
TREM2: Triggering receptor expressed on myeloid cells 2
IPA: Indole-3-propionic acid
LPS: Lipopolysaccharide
SCFAs: Short-chain fatty acids
TMAO: Trimethylamine N-oxide
Kla: Histone lysine lactylation
DG: Dentate gyrus
AHN: Adult hippocampal neurogenesis
imGCs: Immature granule cells
CHI3L1: Chitinase-3-like protein 1
ARIA-E: Amyloid-related imaging abnormalities–edema
FDA: Food and Drug Administration
CSF: Cerebrospinal fluid
CNS: Central nervous system
SGLT2i: Sodium–glucose cotransporter 2 inhibitors
GLP-1: Glucagon-like peptide-1
INI: Intranasal insulin
EMPA: Empagliflozin
FA: Fractional anisotropy
CBF: Cerebral blood flow
LPC: Lysophosphatidylcholine
GPR41: G protein-coupled receptor 41
PA: Propionic acid
MMKD: Mediterranean ketogenic diet
HbA1c: Glycated hemoglobin
IF: Intermittent fasting
S1P: Sphingosine-1-phosphate
HDAC6: Histone deacetylase 6
RTKs: Receptor tyrosine kinases
EPS: Eucommiae cortex polysaccharides
PH-PS: Pseudostellaria heterophylla polysaccharide
PEP: Phyllanthus emblica polysaccharides
24-HC: 24S-hydroxycholesterol
LXRβ: Liver X receptor β
BBB: Blood-brain barrier
NRFs: Nuclear respiratory factors
TFAM: Mitochondrial transcription factor A
mtDNA: Mitochondrial DNA
OXPHOS: Oxidative phosphorylation
DRP1: Dynamin-related protein 1
Fis1: Fission protein 1
ABAD: Aβ-binding alcohol dehydrogenase
TFEB: Transcription factor EB
LAMP1: Lysosomal-associated membrane protein 1
GLUTs: Glucose Transporters
PSD-95: Postsynaptic density protein 95
APPBP2: Amyloid beta precursor protein binding protein 2
mTOR: Mammalian Target of Rapamycin
ApoE: Apolipoprotein E
CD2AP: CD2-associated protein
SEMA4D: Semaphorin 4D‌
